# Autoantibody profiles in patients with immune checkpoint inhibitor-induced neurological immune related adverse events

**DOI:** 10.3389/fimmu.2023.1108116

**Published:** 2023-02-08

**Authors:** Leonie Müller-Jensen, Samuel Knauss, Lorena Ginesta Roque, Christian Schinke, Smilla K. Maierhof, Frederik Bartels, Carsten Finke, Kristin Rentzsch, Claas Ulrich, Raphael Mohr, Werner Stenzel, Matthias Endres, Wolfgang Boehmerle, Petra Huehnchen

**Affiliations:** ^1^ Klinik und Hochschulambulanz für Neurologie, Charité – Universitätsmedizin Berlin, corporate member of Freie Universität Berlin and Humboldt - Universität zu Berlin, Berlin, Germany; ^2^ Berlin Institute of Health at Charité – Universitätsmedizin Berlin, Berlin, Germany; ^3^ Berlin School of Mind and Brain, Humboldt-Universität zu Berlin, Berlin, Germany; ^4^ Clinical Immunological Laboratory Prof. Dr. med. Winfried Stöcker, Groß Grönau, Germany; ^5^ Hauttumorcentrum, Klinik für Dermatologie, Charité – Universitätsmedizin Berlin, corporate member of Freie Universität Berlin and Humboldt-Universität zu Berlin, Berlin, Germany; ^6^ Medizinische Klinik mit Schwerpunkt Gastroenterologie und Hepatologie, Charité – Universitätsmedizin Berlin, corporate member of Freie Universität Berlin and Humboldt-Universität zu Berlin, Berlin, Germany; ^7^ Institut für Neuropathologie, Charité – Universitätsmedizin Berlin, corporate member of Freie Universität Berlin and Humboldt-Universität zu Berlin, Berlin, Germany; ^8^ NeuroCure Cluster of Excellence, Charité – Universitätsmedizin Berlin, corporate member of Freie Universität Berlin and Humboldt-Universität zu Berlin, Berlin, Germany; ^9^ Center for Stroke Research, Charité – Universitätsmedizin Berlin, corporate member of Freie Universität Berlin and Humboldt Universität zu Berlin, Berlin, Germany; ^10^ German Center for Neurodegenerative Diseases (DZNE), partner site, Berlin, Germany; ^11^ German Center for Cardiovascular Research (DZHK), partner site, Berlin, Germany

**Keywords:** immune checkpoint inhibitors, immune related adverse events, neurotoxicity, autoimmunity, neuronal autoantibodies, myositis, paraneoplastic syndromes, cancer immunotherapy

## Abstract

**Background:**

Neurological immune-related adverse events (irAE-n) are severe and potentially fatal toxicities of immune checkpoint inhibitors (ICI). To date, the clinical significance of neuronal autoantibodies in irAE-n is poorly understood. Here, we characterize neuronal autoantibody profiles in patients with irAE-n and compare these with ICI-treated cancer patients without irAE-n.

**Methods:**

In this cohort study (DRKS00012668), we consecutively collected clinical data and serum samples of 29 cancer patients with irAE-n (n = 2 pre-ICI, n = 29 post-ICI) and 44 cancer control patients without irAE-n (n = 44 pre- and post-ICI). Using indirect immunofluorescence and immunoblot assays, serum samples were tested for a large panel of neuromuscular and brain-reactive autoantibodies.

**Results:**

IrAE-n patients and controls received ICI treatment targeting programmed death protein (PD-)1 (61% and 62%), programmed death ligand (PD-L)1 (18% and 33%) or PD-1 and cytotoxic T-lymphocyte-associated protein (CTLA-)4 (21% and 5%). Most common malignancies were melanoma (both 55%) and lung cancer (11% and 14%). IrAE-n affected the peripheral nervous system (59%), the central nervous system (21%), or both (21%). Prevalence of neuromuscular autoantibodies was 63% in irAE-n patients, which was higher compared to ICI-treated cancer patients without irAE-n (7%, p <.0001). Brain-reactive autoantibodies targeting surface (anti-GABA_B_R, -NMDAR, -myelin), intracellular (anti-GFAP, -Zic4, -septin complex), or unknown antigens were detected in 13 irAE-n patients (45%). In contrast, only 9 of 44 controls (20%) presented brain-reactive autoantibodies before ICI administration. However, seven controls developed *de novo* brain-reactive autoantibodies after ICI initiation, therefore, prevalence of brain-reactive autoantibodies was comparable between ICI-treated patients with and without irAE-n (p = .36). While there was no clear association between specific brain-reactive autoantibodies and clinical presentation, presence of at least one of six selected neuromuscular autoantibodies (anti-titin, anti-skeletal muscle, anti-heart muscle, anti-LRP4, anti-RyR, anti-AchR) had a sensitivity of 80% (95% CI 0.52-0.96) and a specificity of 88% (95% CI 0.76-0.95) for the diagnosis of myositis, myocarditis, or myasthenia gravis.

**Conclusion:**

Neuromuscular autoantibodies may serve as a feasible marker to diagnose and potentially predict life-threatening ICI-induced neuromuscular disease. However, brain-reactive autoantibodies are common in both ICI-treated patients with and without irAE-n, hence, their pathogenic significance remains unclear.

## Introduction

Targeting immune checkpoints with monoclonal antibodies against programmed death protein 1 (PD-1), programmed death-ligand 1 (PD-L1) or cytotoxic T-lymphocyte-associated protein 4 (CTLA-4) has been a breakthrough in the treatment of many malignancies (1–3). However, the benefits of immune checkpoint inhibitors (ICIs) are often mitigated by the development of autoimmune phenomena, referred to as immune-related adverse events (irAEs) (4, 5).

In particular, neurological irAEs (irAE-n) are an increasingly recognized complication (6) with mortality rates up to 35% and long-term sequelae in 40-68% of ICI-treated cancer patients (7–11). Common manifestations include encephalitis, myositis, myasthenia gravis (MG) and neuropathies, but every part of the nervous system can be affected (10–15). While the clinical phenotypes of irAE-n are well-described, little is known about the immunological mechanisms and potential biomarkers.

It has been proposed that ICI-induced immune activation may trigger paraneoplastic neurological disorders (PNDs) (16–18). In PNDs, autoantibodies are directed against antigens shared by the tumor and neural tissue and thereby cause – directly or mediated by cytotoxic T cells – off-target reactivity (19). However, it is unknown whether cross-reactivity is the only mechanisms to elicit ICI-induced neurotoxicity (11). As irAE-n can resemble classical, antibody-mediated neurological autoimmune disorders such as MG (20, 21), ICI-induced disruption of immune tolerance may be another decisive factor in the development of irAE-n.

If irAE-n were (partly) antibody-mediated diseases, screening of autoantibodies could help diagnosing and potentially predicting irAE-n. Moreover, treating irAE-n with B cell depletion or plasma exchange could be a valuable addition to the current standard therapy with corticosteroids (22). To further investigate the clinical significance of neuronal autoantibodies in irAE-n, we characterized neuronal autoantibody profiles in cancer patients with irAE-n in comparison to ICI-treated cancer patients without irAEs.

## Methods

### Design, ethics statement and patient consents

This cohort study was registered (DRKS00012668) and approved by the Ethics Committee of Charité Universitätsmedizin Berlin (EA1/099/17 and EA4/219/21). Written informed consent to participate in this study was obtained from all patients prior to any study procedures. The study was conducted at the Charité Universitätsmedizin Berlin between September 2017 and January 2022.

### Patients

We recruited all consecutive patients with irAE-n that met the following inclusion criteria: Cancer patients over 18 years old that (1) received ICI treatment and (2) were diagnosed with an irAE-n according to the consensus criteria of “probable” or “definite” irAE-n as defined previously (23). One additional patient was included who had preexisting MG which deteriorated to a myasthenic crisis after ICI treatment initiation. In one patient, treatment was double-blinded for ICI therapy versus (vs.) placebo, but the patient developed multiple autoimmune phenomena, hence, ICI treatment was highly probable. Three investigators (*LMJ, SK, PH*) confirmed the diagnosis of irAE-n.

**Figure 1:**
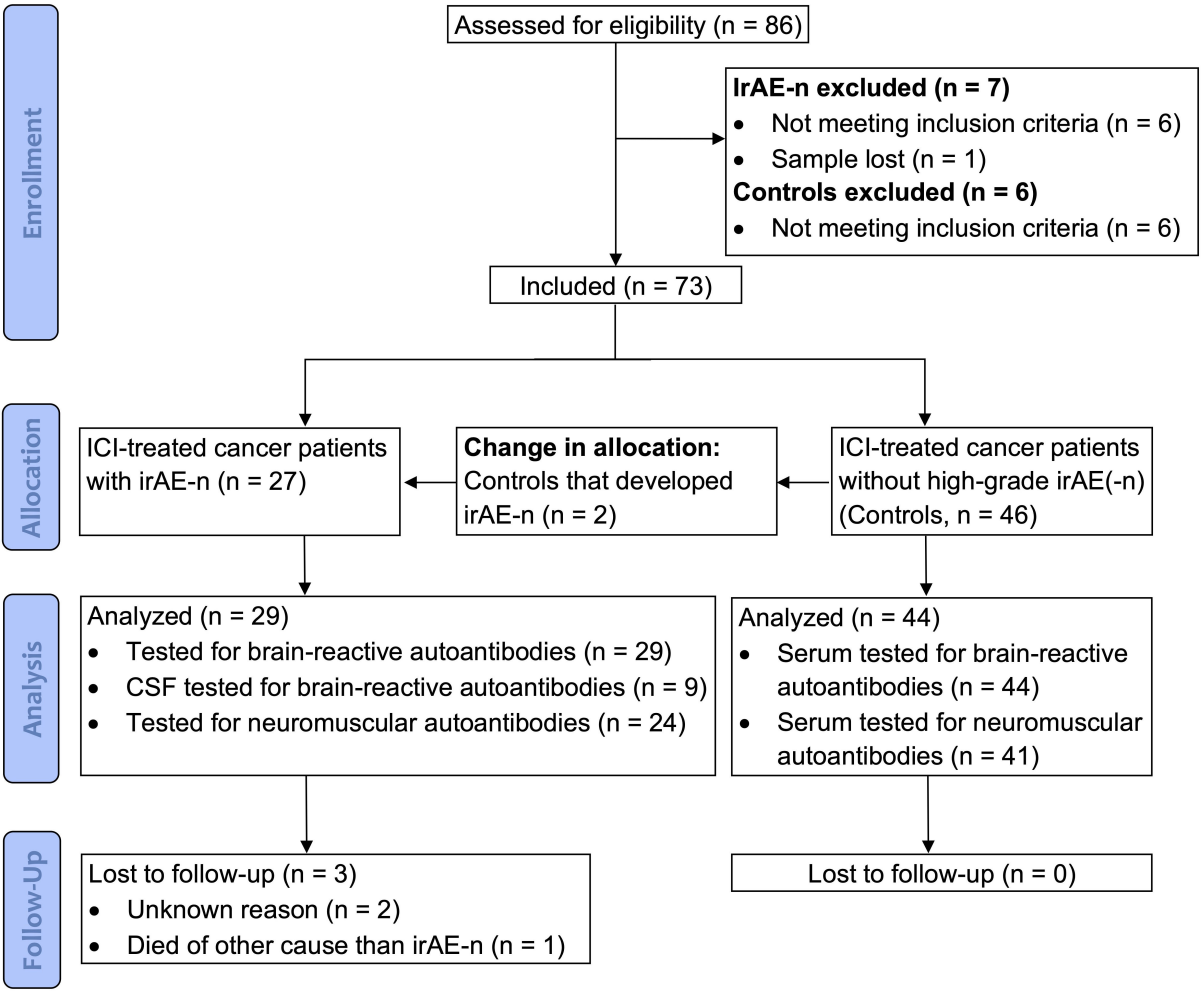
CONSORT diagram. Between September 2017 and January 2022, a total of 86 cancer patients were enrolled for the study. After application of inclusion and exclusion criteria, 73 patients were included. As two patients that were originally allocated to the control cohort developed irAE-n, a total of 29 irAE-n patients and 44 cancer control patients were analyzed. CSF, cerebrospinal fluid; ICI, immune checkpoint inhibitor; irAEs, immune related adverse events; irAE-n, neurological immune related adverse event.

Age-, sex-, ICI- and tumor-matched adult cancer patients that were scheduled for ICI treatment were enrolled as controls. We excluded control patients with previous ICI treatment within the last six months and – as most immunotoxicities occur early after ICI treatment onset - patients that developed any severe (Common Terminology Criteria for Adverse Events [CTCAE] grade ≥3) neurological or non-neurological irAEs within the first three months of treatment. A CONSORT diagram of the study is shown in **Figure 1**.

### Clinical data and outcome

We collected the following clinical variables: Demographics (age, sex), tumor entity, neurological comorbidities, brain metastases (yes or no), ICI type, ICI cycle, type of irAE-n, treatment of irAE-n, other irAE and rechallenge of ICI. Charts and routine care data of all patients were reviewed to score the following outcome parameters retrospectively: CTCAE grade of irAE-n at three months after symptom onset (grade 1 to 5) and steroid treatment at three months after symptom onset (yes or no).

### Neuronal autoantibody testing

Blood serum samples of all patients with irAE-n were collected during the acute disease stage. In addition, nine cerebrospinal fluid (CSF) samples that were assessed during the routine diagnostic workup were included. From controls, blood serum samples were collected before (baseline) and six weeks (IQR, 6-8) after ICI treatment initiation. At the time of the second study visit, patients had received a median of two ICI infusions (IQR, 2-2). As irAE-n are rare and neurological consultation usually takes place when neurological symptoms are already present, assessment of pretreatment samples from irAE-n patients is difficult. However, two patients that were originally allocated to our control cohort developed neurotoxicities (2/46, 4%), therefore, two pretreatment irAE-n samples could be analyzed.

All serum samples were tested for brain-reactive IgG autoantibodies targeting the following antigens: α-amino-3-hydroxy-5-methyl-4-isoxazolepropionic acid receptor 1/2 (AMPAR1/2), amphiphysin, aquaporin 4 (AQP4), RhoGTPase-activating protein 26 (ARHGAP26), ATP1A3, carbonic anhydrase related proteins VIII (CARP VIII), contactin-associated protein-like 2 (CASPR2), collapsin response-mediator protein 5 (CV2/CRMP5), dipeptidyl-peptidase-like protein 6 (DPPX), Flotillin1/2, gamma-aminobutyric-acid A receptor (GABA_A_R), gamma-aminobutyric-acid B receptor (GABA_B_R), glutamic acid decarboxy lase 65 (GAD65), glial fibrillary acidic protein (GFAP), glutamate receptor delta 2 (GluD2), glycine receptor (GlyR), Homer protein homolog 3 (Homer-3), Hu (Anna-1), immunoglobulin LON5 (IgLON5), inositol 1,4,5-trisphosphate receptor 1 (ITPR-1), leucine-rich glioma-inactivated 1 (LGI1), Ma2, metabotropic glutamate receptor 1 (mGluR1), metabotropic glutamate receptor 5 (mGluR5), myelin oligodendrocyte glycoprotein (MOG), myelin, anti-neuroendothelium, neurexin, neurochondrin, N-methyl-D-aspartate receptor (NMDAR), recoverin, Ri (Anna-2), septin complex, Tr (DNER), Yo (PCA-1), and zinc finger 4 (Zic4). In addition, IgA and IgM NMDAR autoantibodies were tested.

During clinical routine, eight of nine available CSF samples were analyzed for IgG autoantibodies directed against the following antigens: Amphiphysin, AQP4, CASPR2, DPPX, GAD65, GABA_B_R, AMPAR1/2, NMDAR, mGluR5, GlyR, LGI1, myelin, CV2/CRMP5, Hu, Ma2, Ri, Tr, Yo. One CSF sample was only tested for eight of the above-mentioned IgG autoantibodies as only small amounts of CSF were available (amphiphysin, CV2/CRMP5, GAD65, Hu, Ma2, Ri, Tr, Yo).

Twenty-four of 29 and 41 of 44 patients with and without irAE-n, respectively, were additionally tested for IgG neuromuscular autoantibodies targeting the following antigens: Acetylcholine receptor (AchR), heart muscle, skeletal muscle, lipoprotein receptor-related protein 4 (LRP4, only tested in n = 40 controls), myelin-associated glycoprotein (MAG), muscle-specific tyrosine kinase (MuSK), P/Q-type voltage-gated calcium channel (P/Q VGCC, only tested in n = 40 controls), ryanodine receptor (RyR, only tested in n = 40 controls), SRY-related HMG-box 1 (SOX1), and titin. Specifications of all analyzed autoantibodies are described in **Supplemental Table 1**.

Detection of brain-reactive autoantibodies was performed using commercial assays (all EUROIMMUN Medizinische Labordiagnostika AG, Germany) including cell-based assays (CBA) and immunohistochemistry (IHC) of frozen brain tissue (rat hippocampus, rat cerebellum, and monkey cerebellum). To that end, indirect immunofluorescence using BIOCHIP mosaics™ was performed as described previously (24). To confirm CBA or IHC results for intracellular antigens, immunoblot assays (EUROLINE) were additionally performed. If autoantibodies were detected *via* IHC, but CBA and EUROLINE were negative, they were considered autoantibodies of unknown reactivity.

Neuromuscular autoantibodies were tested with the following commercial assays (all Labor Berlin GmbH, Germany): Anti-titin and anti-SOX1 autoantibodies using line assays; anti-LPR4, anti-skeletal muscle and anti-heart muscle autoantibodies using indirect immunofluorescence; anti-MAG, anti-MuSK, and anti-AchR autoantibodies using enzyme linked immunosorbent assays; anti-RyR autoantibodies using western blot and anti-P/Q VGCC autoantibodies using radioimmunoassays. To enhance sensitivity, we considered weakly positive or borderline positive results as positive.

### Statistical analysis

Continuous and categorical variables were reported as median (interquartile range, [IQR]) and numbers (percentage), respectively. Group differences of unpaired categorical data were analyzed using the Chi-squared test or Fisher’s exact test. Comparison of paired categorical data (baseline vs. six weeks after ICI treatment onset) was conducted using the McNemar test. Group differences of continuous variables were calculated using the Mann-Whitney U test. Sensitivity, specificity, and 95% confidence intervals (CI) were calculated using the “EpiR” R package to investigate the diagnostic accuracy for specific autoantibodies. We considered an alpha-level of ≤ 0.05 as statistically significant. Graphpad Prism (version 7) and RStudio (version 2022.02.3 + 492 “Prairie Trillium”) were used for graph illustration and statistical analysis.

## Results

Twenty-nine cancer patients who were diagnosed with irAE-n and 44 ICI-treated cancer patients without high-grade (CTCAE ≥ 3) irAEs were included. Patients’ demographics and clinical characteristics are summarized in **Table 1**.

**Table 1:** Characteristics of ICI-treated cancer patients with and without neurological irAEs.

Characteristic	ICI-treated cancer patients without irAEs (controls) (n = 44)	Patients with irAE-n, all (n = 29)	Patients with irAE-n, brain-reactive ab+ (n = 13)	Patients with irAE-n, brain- reactive ab- (n = 16)	Patients with irAE-n, Neuromuscular ab+ (n = 15)	Patients with irAE-n, Neuromuscular ab- (n = 9)
**Female**	15/44 (34)	8/29 (28)	4/13 (31)	4/16 (25)	3/15 (20)	3/9 (33)
**Age at onset, y**	68 (60-76)	65 (61-74)	61 (61-68)	69 (61-77)	63 (60-76)	65 (61-68)
**Neoplasm**						
Melanoma	24/44 (55)	16/29 (55)	7/13 (54)	9/16 (56)	9/15 (60)	4/9 (44)
NSCLC/SCLC	5/44 (11)	4/29 (14)	1/13 (8)	3/16 (19)	3/15 (20)	1/9 (11)
Hepatocellular carcinoma	11/44 (25)	2/29 (7)	2/13 (15)	0/16) (0)	2/15/13)	0/9 (0)
Gastric cancer/OGJ	0/44 (0)	2/29 (7)	1/13 (8)	1/16 (6)	0/15 (0)	2/9 (22)
Other	4/44 (9)^a^	5/29 (17)^b^	2/13 (15)	3/16 (19)	1/15 (7)	2/9 (22)
**Brain metastases**	5/44 (11)	3/29 (10)	1/13 (8)	2/16 (13)	0/15) (0)	3/9 (33)
**Neurological comorbidity**	9/44/20)^c^	3/29 (10)^d^	0/13 (0)	3/16 (19)	2/15 (13)	1/9 (11)
**Non-neurological irAEs**	9/44 (20)^e^	18/29 (62)	8/13 (62)	10/16 (63)	11/15 (73)	5/9 (56)
**ICI therapy**						
PD-1	26/42 (62)	17/28 (61)	7/13 (54)	10/15 (67)	10/15 (67)	4/9 (44)
PD-L1	14/42 (33)	5/28 (18)	4/13 (31)	1/15 (7)	3/15 (20)	2/9 (22)
ICI combination (PD-1 + CTLA-4)	2/42 (5)	6/28 (21)	2/13 (15)	4/15 (27)	2/15 (13)	3/9 (33)
**IrAE-n**						
Encephalitis	n/a	7/29 (24)	3/13 (23)	4/16 (25)	3/15 (20)	4/9 (44)
Hypophysitis	n/a	5/29 (17)	2/13 (15)	3/16 (19)	2/15 (13)	1/9 (11)
Neuropathy (incl. GBS)	n/a	13/29 (41)	7/13 (54)	6/16 (38)	7/15 (47)	3/9 (33)
Myositis/ Myopathy/ Myocarditis	n/a	11/29 (38) 1/29 (3) isolated myocarditis	4/13 (31)	7/16 (44) 1/16 (6) isolated myocarditis	8/15 (53) 1/15 (7) isolated myocarditis	3/9 (33)
Myasthenia gravis	n/a	4/29 (14)	2/13 (15)	2/16 (13)	4/15 (27)	0/9 (0)
≥ 2 irAE-n^g^	n/a	10/29 (34)	5/13 (38)	5/16 (31)	6/15 (40)	3/9 (33)
**Brain-reactive autoantibodies**	before ICI: 9/44 (20) after ICI: 15/44 (34)	13/29 (45)	13/13 (100)	0/16 (0)	6/15 (40)	6/9 (67)
**Neuromuscular autoantibodies**	before ICI: 7/41 (17) after ICI: 3/41(7)	15/24 (63)	6/12 (50)	9/14 (64)	15/15 (100)	0/9 (0)
**Onset after first ICI cycle, weeks**	n/a	12 (7-24)	15 (9-24)	12 (5-21)	9 (5-30)	15 (10-24)
**Highest CTCAE grade of irAE-n**	n/a	3 (3-4)	3 (2-3)	3 (3-4)	3 (3-4)	3 (3-4)
**Treatment**						
Corticosteroids	n/a	27/29 (93)	12/13 (92)	15/16 (94)	15/15 (100)	8/9 (89)
IVIG	n/a	7/29 (24)	3/13 (23)	4/16 (25)	5/15 (33)	1/9 (11)
Plasma exchange	n/a	2/29 (7)	0/13 (0)	2/16 (13)	1/15 (7)	1/9 (11)
**Unfavorable outcome (CTCAE ≥ 3 at 3m)**	n/a	13/29 (45)^h^	4/13 (31)	9/16 (56)	8/15 (53)	4/9 (44)
**Corticosteroids at 3m**	n/a	22/24 (92)	10/11 (91)	12/13 (92)	13/13 (100)	6/7 (86)
**Lethal outcome of irAE-n**	n/a	3/29 (10)	1/13 (8)	2/16 (13)	2/11 (18)	1/9 (11)

Values are median (interquartile range, IQR) or n (%). ab, autoantibody; CTCAE, Common Terminology Criteria for Adverse Events; CSF, cerebrospinal fluid; CTLA-4, cytotoxic T-lymphocyte-associated protein 4; GBS, Guillain-Barré syndrome; ICI, immune checkpoint inhibitor**;** ICU, intensive care unit; irAEs, immune related adverse events; IVIG, intravenous immunoglobulin therapy; n/a, not applicable; NSCLC, non-small cell lung cancer; OGJ, oesophagogastric junctional adenocarcinoma; PD-1, programmed cell death protein 1; PD-L1, programmed death-ligand 1; SCLC, small cell lung cancer; y, years; 3m, three months. ^a^ = basal-cell carcinoma, Merkel cell carcinoma, squamous cell carcinoma. ^b^ = cholangiocarcinoma, Hodgkin lymphoma, Merkel cell carcinoma, prostate cancer, urothelial carcinoma. ^c^ = five cases of previous stroke, one case of previous stroke and epilepsy, one case of previous stroke and meningioma, one case of chemotherapy-induced polyneuropathy, one case of previous vestibular neuritis. ^d^ = one case of previous stroke, one case of Korsakoff syndrome, one case of preexisting myasthenia gravis with anti-AchR and anti-titin autoantibodies. ^e^ = patients with high-grade irAE (CTCAE grade ≥ 3) were excluded. ^f^ = two patients in the control cohort and one patient in the irAE-n group were blinded for ICI-type (nivolumab +/- ipilimumab). ^g^ = one patient had three irAE-n: myositis, neuropathy, encephalitis ^h^ = in two patients CTCAE grade was scored at one month, as patients were lost to follow-up afterwards. In one patient, last CTCAE grade was evaluated, as the patient died of sepsis one week after onset of irAE-n.

### Autoantibody profiles in ICI-treated cancer patients without (non-)neurological irAEs

#### Neuromuscular autoantibodies

**Figure 2:**
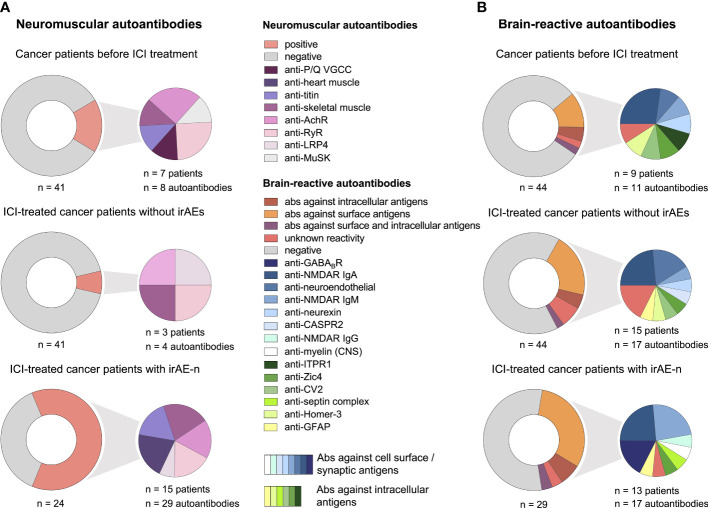
Serum autoantibody profiles in cancer patients with irAE-n and controls. **(A)** Frequency and specification of neuromuscular autoantibodies of 41 cancer patients before (*upper left)* and six weeks after (*middle left*) ICI treatment initiation who did not develop high-grade (= CTCAE ≥ 3) irAEs and 24 ICI-treated cancer patients with irAE-n (*lower left*). **(B)** Frequency and specification of brain-reactive autoantibodies of 44 cancer patients before (*upper right)* and six weeks after (*middle right*) ICI treatment initiation who did not develop high-grade (CTCAE ≥ 3) irAEs and 29 ICI-treated cancer patients with irAE-n (*lower right)*. Ab, autoantibody; AchR, acetylcholine receptor; CASPR2, contactin-associated protein-like 2; GABA_B_R, gamma-aminobutyric-acid B receptor; GFAP, glial fibrillary acidic protein; Homer-3, Homer protein homolog 3; ICI, immune checkpoint inhibitor; irAEs, immune related adverse events; irAE-n, neurological immune related adverse event; ITPR1, inositol 1,4,5-trisphosphate receptor 1; LRP4, low-density lipoprotein receptor-related protein 4; MuSK, muscle-specific tyrosine kinase; NMDAR, N-methyl-D-aspartate receptor; P/Q VGCC, P/Q-type voltage gated calcium channel; RyR, ryanodine receptor; Zic4, zinc finger 4.

Prevalence of neuromuscular autoantibodies was 17% (7/41 tested patients) in control patients before ICI treatment (**Figure 2A**). Of these, three were only borderline positive; one for anti-PQ VGCC autoantibodies (31 pmol/l; upper reference limit, 30 pmol/l) and two for anti-AchR autoantibodies (0.4 nmol/l; upper reference limit, 0.4 nmol/l). In contrast, six weeks after ICI treatment initiation (equal to two ICI infusions) neuromuscular autoantibodies were detected in only three controls (7%, p = <.0001): In t wo of the seven patients that were initially tested positive a nd in one additional patient with *de novo* anti-LRP4 autoantibodies.

Interestingly, one of these patients presented anti-titin and anti-RyR autoantibodies pre- and post-ICI treatment and reported “muscle weakness” and “exhaustion” at the last routine follow up. Unfortunately, the patient was in palliative care, therefore no further examinations were initiated.

#### Brain-reactive autoantibodies

Before ICI treatment, nine of 44 control patients (20%) were tested positive for brain-reactive autoantibodies (**Figure 2B**). In two patients two different brain-reactive autoantibodies were detected (anti-Zic4 [1:32000] and anti-CV2/CRMP5 [1:32]; anti-Homer-3 [1:1000] and IgA anti-NMDAR [1:100]). Two of nine patients with brain-reactive autoantibodies had diagnosed brain metastases, none had history of herpetic encephalitis or autoimmune disease of the central nervous system (CNS) as potential triggers of brain-reactive autoantibodies.

Interestingly, we noticed a *de novo* development of brain-reactive autoantibodies after ICI treatment initiation: Seven patients who were initially tested negative had developed brain-reactive autoantibodies after two ICI infusions (**Figure 2B**). In contrast, only one patient with brain-reactive autoantibodies at baseline (ITPR3, 1:320) was tested negative at the second visit, so the prevalence of brain-reactive autoantibodies was higher after ICI treatment initiation compared to baseline (34%, p =.002). Of patients with *de novo* autoantibodies, one (14%) had autoantibodies against intracellular antigens (anti-GFAP [1:320]), four (57%) had autoantibodies against surface antigens (IgM anti-NMDAR [1:320]; anti-CASPR2 [1:32]; anti-neuroendothelial autoantibodies [1:1000 and 1:320]) and two (29%) had autoantibodies of unknown reactivity. One additional patient showed seroconversion from IgM anti-NMDAR (1:10) to IgA anti-NMDAR (1:10). *De novo* Development of brain-reactive autoantibodies was not associated with a specific neoplasm (four cases of melanoma, two cases of non-small cell lung cancer [NSCLC], one case of hepatocellular carcinoma), ICI type (four cases with nivolumab treatment [PD-1 inhibitor], three cases with atezolizumab treatment [PD-L1 inhibitor]), or neurological symptoms.

### Autoantibody profiles in ICI-treated cancer patients with irAE-n

Of 29 patients, irAE-n affected the CNS in six (encephalitis, hypophysitis), the peripheral nervous system (PNS) in 17 (neuropathy, myositis, MG) and both in another six patients (**Table 1**). A detailed cohort description can be found in **Supplemental Table 2**.

#### Neuromuscular autoantibodies

The prevalence of neuromuscular autoantibodies was 63% (15/24; **Figure 2A**) in patients with any irAE-n and 80% (12/15) in patients with either ICI-induced myositis, myocarditis, or MG. Compared to controls, neuromuscular autoantibodies were significantly more common in patients with irAE-n (63% vs. 7%; p = <.0001).

Detected autoantibodies in patients with myositis, myocarditis or MG were anti-heart muscle (six cases), anti-skeletal muscle (six cases), anti-titin (five cases), anti-RyR (four cases), anti-AchR (three cases), and anti-LRP4 (one case) autoantibodies. Eight patients (67%) had more than one neuromuscular autoantibody (**Supplemental Table 2**).

**Figure 3:**
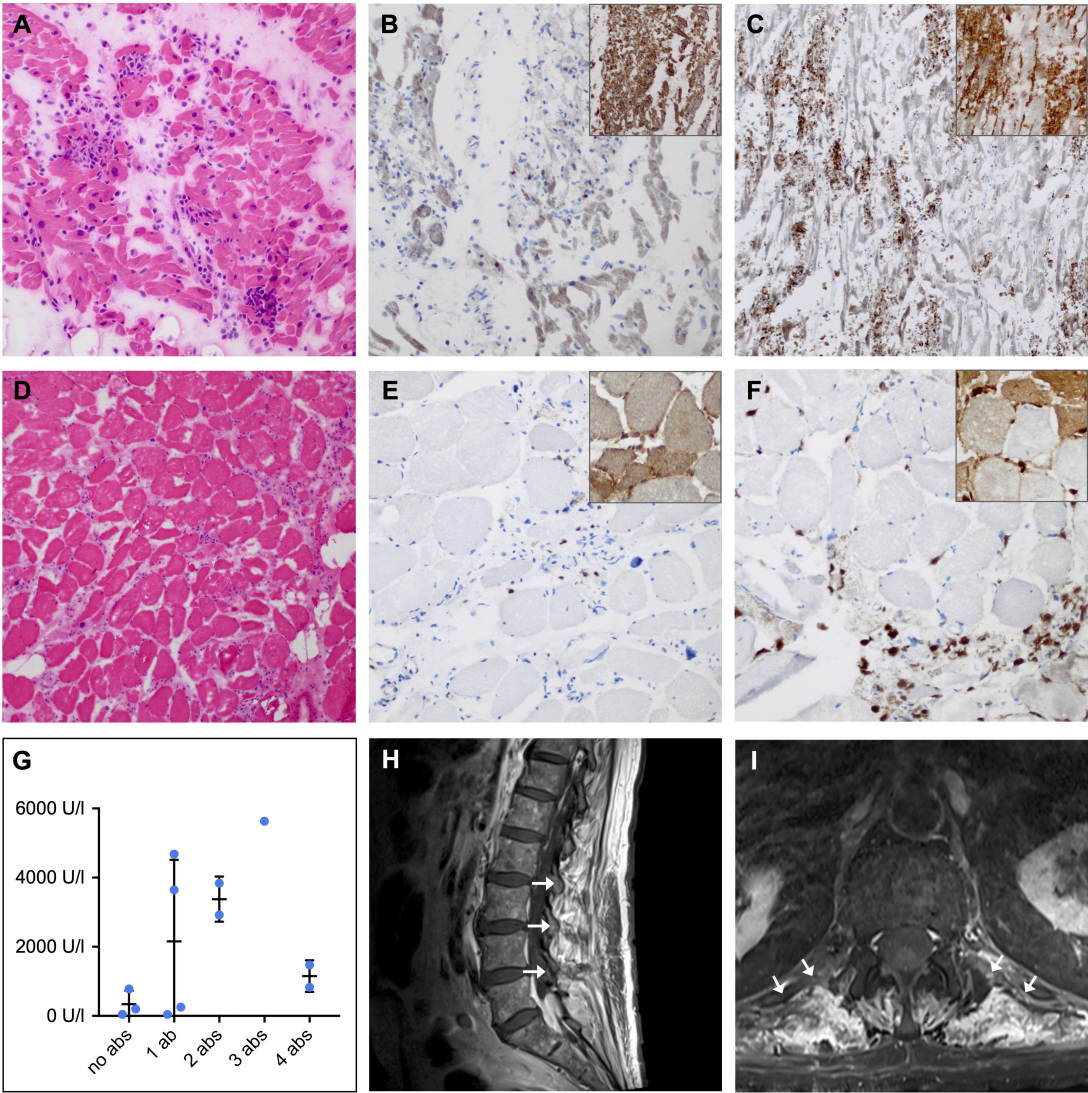
Histopathological and clinical findings in patients with ICI-induced myositis. **(A-F)** Hematoxylin-eosin (HE) and immunohistochemical staining in a female patient with fatal ICI-induced myositis and myocarditis and positive anti-titin, anti-heart muscle and anti-skeletal muscle autoantibodies. Frozen sections of HE-stained **(A)** cardiac muscle **(D)** and skeletal muscle showing necrotic myofibers and lymphocyte infiltration (original magnification x 100). CD8 (large panel, original magnification x 200) and major histocompatibility complex (MHC) class I (inset, original magnification x 100) staining revealing cytotoxic T cell invasion and sarcolemmal overexpression of MHC class I by **(B)** cardiac and **(E)** skeletal muscle fibers. CD68 (large panel, original magnification x 200) and MHC class II (inset, original magnification x 100) staining showing macrophage invasion and sarcolemmal as well as sarcoplasmic overexpression of MHC class II by **(C)** cardiac and **(F)** skeletal muscle fibers. **(G)** Comparison of peripheral blood creatine kinase (CK) levels in patients with ICI-induced myositis shows a trend towards higher CK levels in patients with multiple neuromuscular autoantibodies compared to patients without neuromuscular autoantibodies. **(H)** Sagittal and **(I)** axial contrast-enhanced magnetic resonance imaging of the lumbar spine with T1-weighted turbo spin echo sequence showing paravertebral contrast enhancement (*arrows*) as a sign of muscle edema in a patient with ICI-induced myositis and myasthenia. Ab, autoantibody; ICI, immune checkpoint inhibitor; U/l, units per liter.

As two patients that were originally allocated to our control cohort developed irAE-n (2/46, 4%), two pretreatment irAE-n samples could be analyzed. One sample revealed preexisting, high-titer anti-titin, anti-heart muscle and anti-skeletal muscle autoantibodies in an 86-year-old female patient with melanoma, who died of fulminant myocarditis and myositis after one ICI infusion (patient no. 11 in **Supplemental Table 2**). Autopsy was performed and demonstrated invasion of predominantly macrophages and cytotoxic T cells as well as an overexpression of major histocompatibility complex class I and II in necrotic cardiac (**Figures 3A-C**) and skeletal muscle tissues (**Figures 3D-F**). The other sample was from a 52-year-old female, who was diagnosed with peripheral facial nerve palsy. Pre-ICI treatment no neuromuscular autoantibodies were detected, but after two ICI cycles the patient was tested positive for low-titer anti-AchR autoantibodies (patient no. 29 in **Supplemental Table 2**).

To investigate the diagnostic accuracy of neuromuscular autoantibodies in patients with ICI-induced myositis, myocarditis, or MG, we selected the six most abundant autoantibodies (anti-heart muscle, anti-titin, anti-skeletal muscle, anti-RyR, anti-AchR and anti-LRP4) and compared detection of either of these autoantibodies between cancer patients with and without ICI-induced neuromuscular disease. Sensitivity and specificity were high with 80% (95% CI 0.52-0.96) and 88% (95% CI 0.76-0.95), respectively. The positive and negative predictive values were 67% (95% CI 0.41-0.87) and 94% (95% CI 0.82-0.99), respectively. Six of 65 ICI-treated cancer patients (9%) had positive neuromuscular autoantibodies but no diagnosed myositis, myocarditis, or MG. Three of these six patients had ICI-induced neuropathy (patient no. 23, no. 28, and no. 29 in **Supplemental Table 2**). Conversely, in three patients with myopathy no neuromuscular autoantibodies were detected. However, all of them presented only mild symptoms; one with normal creatine kinase [CK] levels and all with only discrete myopathic changes in the electromyographic activity.

Interestingly, CK levels tended to be higher in patients with myositis or myocarditis and multiple neuromuscular autoantibodies compared to patients with none or only one neuromuscular autoantibody (**Figure 3G**). Regarding clinical characteristics such as age, sex, neoplasm, or ICI type, we did not observe differences between irAE-n patients with and without neuromuscular autoantibodies (**Table 1** and **Supplemental Table 3**).

#### Brain-reactive autoantibodies

**Figure 4:**
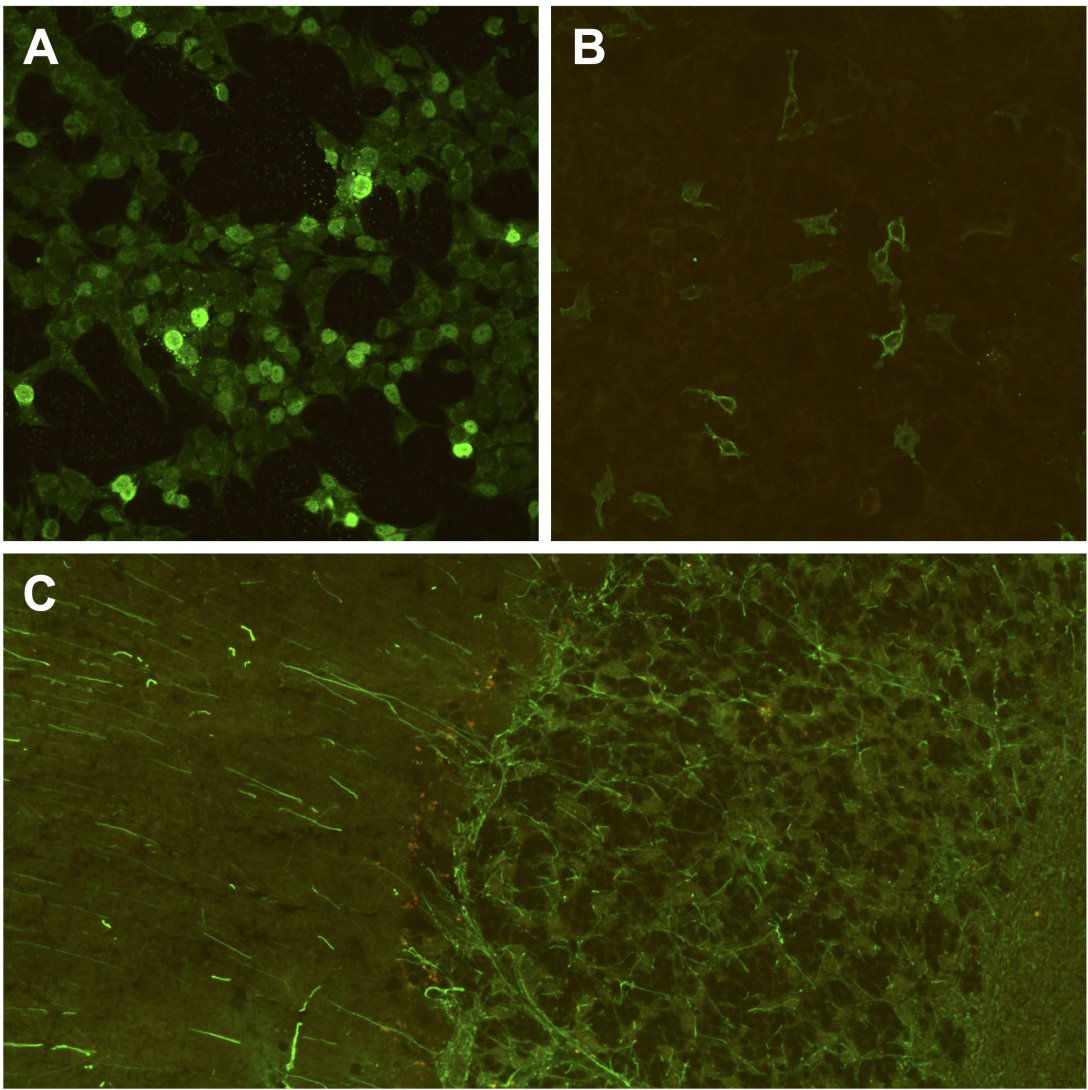
Brain-directed autoantibodies in ICI-treated cancer patients with irAE-n. **(A, B)** Cell-based assays of fixed recombinant HEK293-cells (original magnification x 200) expressing single neuronal antigens showing anti-Zic4 autoantibodies (1:100 dilution; **A**) and anti-GABA_B_R autoantibodies (1:10, **B**) in a patient with small cell lung cancer and Guillain-Barré syndrome (GBS) after pembrolizumab treatment. However, anti-ganglioside autoantibodies, which are associated with GBS, were tested negative during clinical routine. **(C)** Tissue-based assay using indirect immunofluorescence screening on monkey cerebellum sections (original magnification x 200) showing anti-GFAP autoantibodies (1:100 dilution) in a melanoma patient with encephalitis after treatment with ipilimumab and nivolumab. GABA_B_R, gamma-aminobutyric-acid B receptor; GFAP, glial fibrillary acidic protein; ICI, immune checkpoint inhibitor; irAE-n, neurological immune related adverse event; Zic4, zinc finger 4.

The frequency of brain-reactive autoantibodies in patients with irAE-n was 45% (13/29), which was only slightly higher compared to ICI-treated cancer patients without high-grade irAEs (45% vs. 34%, p = .36; **Figure 2B** and **Supplemental Table 2**). In four patients two different brain-reactive autoantibodies were detected (IgG anti-NMDAR [1:10] and anti-GABA_B_R [1:10]; anti-Zic4 [1:32000] and anti-GABA_B_R [1:10, **Figures 4A, B**]; IgA anti-NMDAR [1:32] and IgM anti-NMDAR [1:100]; IgA anti-NMDAR [1:320] and IgM anti-NMDAR [1:10]). We did not find any brain-reactive autoantibodies in nine additionally tested CSF samples (**Supplemental Table 2**).

The two analyzed pretreatment samples revealed *de novo* anti-GABA_B_R autoantibodies in a female patient with peripheral facial nerve palsy (while IgG anti-NMDAR autoantibodies were detected both pre- and post-ICI treatment; patient no. 29 in **Supplemental Table 2**) and no brain-reactive autoantibodies (neither pre- nor post-ICI treatment) in a female patient with myositis (patient no. 11 in **Supplemental Table 2**).

Comparison of clinical syndrome and autoantibody type indicated a low correlation: Of seven patients with ICI-induced encephalitis three were tested positive for brain-reactive autoantibodies (43%), but only one patient (14%) presented symptoms that matched the autoantibody-associated syndrome. This 81-year-old male patient (patient no. 4 in **Supplemental Table 2**) presented with disorientation, confusion and memory deficits and was tested positive for anti-GFAP autoantibodies in the serum (1:320; **Figure 4C**). CSF showed mononuclear pleocytosis with 51 cells per microliter and CSF protein of 1234 mg/l. Brain MRI was normal. The other two patients with encephalitis had IgA anti-NMDAR autoantibodies (patient no. 7 in **Supplemental Table 2**) and IgA and IgM anti-NMDAR autoantibodies (patient no. 9 in **Supplemental Table 2**). All other patients with irAE-n and positive autoantibodies had PNS manifestation or hypophysitis without any association to brain-reactive autoantibodies. There were no significant differences between patients with and without brain-reactive autoantibodies regarding age, sex, neoplasm, or ICI type (**Table 1** and **Supplemental Table 3**).

### Neuronal autoantibodies and outcome of irAE-n

Three patients (10%) died of the irAE-n (**Table 1**). Median CTCAE grade at three months after symptom onset was 2 (IQR, 2-3). Of 24 patients with available data, 22 (92%) still received steroid treatment three months after symptom onset. Unfavorable outcome (defined as CTCAE ≥ 3 at three months after symptom onset) was observed in 13 patients in total (45%), in four of 13 patients with brain-reactive autoantibodies (31%) and in nine of 16 patients without brain-reactive autoantibodies (56%, p =.17). Likewise, presence of neuromuscular autoantibodies was not associated with unfavorable outcome (53% vs. 44% for patients with and without neuromuscular autoantibodies, respectively, p = .67). Two patients with myositis and/or MG (patient no. 3 and patient no. 22 in **Supplemental Table 2** and **Figures 3H, I**) were rechallenged with ICI treatment during follow-up, both developed a flare of myositis.

## Discussion

In this study, we characterized neuronal autoantibody profiles of 73 cancer patients with and without ICI-induced irAE-n and thereby provide new insights into ICI-induced autoimmunity: (1) Neuronal autoantibodies are common in patients with irAE-n, (2) sensitivity and specificity of neuromuscular autoantibodies is high for the diagnosis of ICI-induced myositis, myocarditis and MG, (3) ICI treatment may induce the production of brain-reactive autoantibodies, however, (4) development of brain-reactive autoantibodies is not necessarily associated with neurological symptoms.

With an incidence of 0.7-1% and a mortality rate of up to 35%, ICI-induced myositis and MG are among the most common and most threatening irAE-n (10, 14). Clinical presentation may be confusing with symptoms typically attributable to different disease entities (e.g., dropped head syndrome), complicating diagnosis and treatment (25, 26). Anti-AchR autoantibodies and striational autoantibodies (anti-titin, anti-heart muscle, and anti-skeletal muscle autoantibodies) have been described in both ICI-induced MG and myositis (21, 26–30), while myositis-specific autoantibodies (e.g., anti-Jo-1, anti-Mi-2 or anti-signal recognition particle autoantibodies) are mostly negative (31, 32). In a cohort of 24 patients with ICI-induced myositis, striational autoantibodies were found to be most common (8/17 [47%] striational autoantibodies vs. 3/17 [18%] for anti-AchR autoantibodies and 0/13 [0%] for myositis-specific autoantibodies, respectively) (32). We detected an even higher prevalence (80%) of neuromuscular autoantibodies in patients with ICI-induced neuromuscular disease and demonstrate that these autoantibodies are rare (7%) in ICI-treated patients without neurotoxicity.

Nevertheless, the immunological relevance of neuromuscular autoantibodies in these fulminant toxicities is still poorly understood. While anti-AchR autoantibodies are directly pathogenic in classical MG (33, 34), striational autoantibodies are more likely to be an immunological epiphenomenon because of their intracellular localization (35, 36). In fact, in our patient with fatal myositis and preexisting striational autoantibodies, CD8+ T cell infiltration and enhanced major histocompatibility complex expression was detected in both cardiac and skeletal muscle tissue. Others, too, reported invasion of cytotoxic T-cells and macrophages into muscular tissue of patients with anti-PD-1/PD-L1-associated myopathy and discovered expression of muscle-specific transcripts in tumor specimens *via* whole-transcriptome sequencing (31, 37). Intriguingly, Axelrod et al. recently identified α-myosin as one autoantigen which is targeted by self-reactive T cells in ICI-induced myocarditis (38). Consequently, it is possible that in patients with ICI-induced neuromuscular disease and striational autoantibodies, T cell-mediated mechanisms dominate, while in those with anti-AchR autoantibodies the autoantibody itself is pathogenic. As this may have therapeutical implications (T cell targeted therapies vs. B cell depletion or plasma exchange), future studies are warranted to investigate optimal treatment approaches in patients with ICI-induced autoantibody-positive neuromuscular disease.

Even though the pathogenicity of neuromuscular autoantibodies is only partly elucidated, their potential as diagnostic markers is evident. Whether autoantibodies are also feasible to predict irAE-n, however, is unclear. ICI-induced myositis and MG may occur early after treatment onset, with some patients developing symptoms after the first ICI infusion (27, 31). In our cohort, one patient presented myositis within one week after first ICI cycle. Early occurrence of irAE-n could point towards a preexisting subclinical autoimmune condition, as the priming of antigen-specific T cells requires days to weeks (39, 40). This hypothesis is supported by the detection of preexisting striational autoantibodies in four other patients with ICI-induced myositis, which all developed symptoms after the first ICI infusion (n = 1) or within 15 days after ICI treatment initiation (n = 1 after 8 days, n = 2 after 15 days), respectively (27, 41). Considering other irAEs, a recent study with 137 patients reported a strong association of preexisting rheumatic autoantibodies and anti-thyroid autoantibodies with the development of irAEs and improved tumor response (42). A study that systematically investigates the diagnostic performance of common rheumatic autoantibodies (antinuclear autoantibodies, rheumatoid factor, and antineutrophil cytoplasmic autoantibodies) to predict irAEs is currently recruiting (43). As neuromuscular irAE-n are particularly threatening immunotoxicities, screening of neuromuscular autoantibodies prior to ICI treatment may also be beneficial.

In addition, identifying clinical risk factors that are associated with ICI-induced neuromuscular disease is paramount. In our cohort, neuromuscular autoantibodies occurred in the context of both PD-1/PD-L1 monotherapy and ICI combination treatment and was not associated with specific malignancies or outcome. However, development of self-reactive T cells may be dependent on patient-specific characteristics such as tumor antigen repertoire and HLA-type (44, 45), so future research should emphasize histopathological and genetic studies.

We detected brain-reactive autoantibodies in 45% of ICI-treated cancer patients with irAE-n. Similarly, in a clinical cohort of 13 patients with irAE-n, 54% of patients had detectable neuronal autoantibodies (30). In contrast, recent studies showed a significantly lower prevalence of brain-reactive autoantibodies in cancer patients overall (25%) and healthy controls (2.5%) (46). However, most autoantibody-positive patients in our cohort did not develop the clinical syndrome typically associated with the detected autoantibody. Only one patient had symptoms consistent with GFAP-associated autoimmunity. Likewise, Sechi et al. reported that the minority of autoantibody-positive patients with irAE-n (6 of 31) presented as “classical” PNDs (30).

On the other hand, ICI-induced PNDs with typical clinical presentation and autoantibodies have been described (18) and it is known that preexisting PNDs worsen after ICI administration (30). Therefore, the pathogenicity of brain-reactive autoantibodies and the role of cancer neoantigens in patients with irAE-n of the CNS is being discussed controversially (11, 16).

In our cohort, 11 of 13 patients with irAE-n and detectable autoantibodies had autoantibodies against myelin, septin complex, NMDAR (IgA or IgM isotype), or an unknown antigen. These autoantibodies are not tested regularly in commercially available assays and their significance is unclear. In fact, in a large cohort of 300 patients, only IgG anti-NMDAR autoantibodies were associated with anti-NMDAR encephalitis, while IgA and IgM anti-NMDAR autoantibodies occurred nonspecifically in diseases like stroke or dementia (47). Otherwise, IgA anti-NMDAR autoantibodies have been linked to progressive cognitive dysfunction, a relevant decrease in NMDAR levels, and prominent changes in NMDAR-mediated currents (48). A strong association of cognitive impairment and brain-reactive autoantibodies – including IgA anti-NMDAR autoantibodies – has been shown in patients with lung cancer and melanoma (24, 46). As the patients in our cohort did not receive systematic neuropsychological testing, it is possible that more subtle cognitive impairment was missed or attributed to comorbidities within primary care.

Interestingly, we discovered a *de novo* development of brain-reactive but not neuromuscular autoantibodies in seven patients after anti-PD-1 or anti-PD- L1 administration. Even though four of seven patients (57%) developed surface-reactive autoantibodies, which can have a direct pathogenic effect on neuronal antigens, none of the patients were later diagnosed with irAE-n. Even though the specific mechanisms remain elusive, these findings indicate ICI-induced and possibly organ-specific disruption of immune tolerance also in ICI-treated patients without clinical signs of autoimmunity. Future studies with larger cohorts are needed to systematically assess cognitive function in antibody-positive patients and to identify risk factors for the clinical emergence of PNDs.

The mortality of irAE-n was 10% in our cohort. Ninety-two percent of patients still received steroids at three months after onset, implying a high risk for long-term adverse events like Cushing’s syndrome, secondary hypopituitarism and reduced antitumor immunity. A recent study with 387 melanoma patients reported chronic irAEs in 43% of patients (49). Together with endocrinopathies, neurotoxicities were most likely to transition to chronic illness. Hence, clinicians should be aware of irAE-n and initiate careful neurological evaluation in all cases of new-onset muscle weakness, dysphagia or dysarthria, hypo- or dysesthesia, disorientation, or cognitive impairment in ICI-treated patients.

In our cohort, every third patient with irAE-n had multiple neurotoxicities. Neuromuscular autoantibodies were also detected in patients with ICI-induced neuropathy. It is possible, that irAE-n - and in particular irAE-n of the PNS - constitute a disease continuum, so clinicians should be vigilant about overlap syndromes with potentially inferior prognosis. In contrast to a previous study on ICI-induced encephalitis (50), however, we and others (30) did not observe an association between neuronal autoantibodies and poor outcome. Taking into account that autoantibody-positive irAE-n might respond to non-steroid treatment approaches (e.g., plasma exchange, intravenous immune globulins, or rituximab), which are less likely to mitigate antitumor response (7, 20, 22), positive autoantibody status may in fact be associated with better outcome in certain cases.

### Strengths and limitations

To our knowledge, this is the first systematic assessment of a large panel of neuronal autoantibodies in cancer patients with and without irAE-n before and after ICI treatment initiation, providing valuable information for clinicians and researchers. Together with others (27, 42), our findings provide the groundwork for pretreatment autoantibody screening to improve ICI safety.

However, several limitations must be acknowledged. First, the small sample size does not allow for definite conclusions on the prevalence of neuronal autoantibodies in ICI-treated cancer patients. Second, our observation time was only three months. We do not know whether controls developed irAE-n beyond that period. Although most irAE-n occur early, some patients develop symptoms after one year of ICI treatment (21, 51), hence, the short follow up time is a weakness. Third, irAE-n are still a diagnosis of exclusion. Even though we used the most comprehensive diagnostic criteria (22), misdiagnosis or false-negative cases that were excluded are possible. Lastly - but most importantly - only two pretreatment samples from patients with irAE-n were available, which is explained by the low frequency of these adverse events. Therefore, we provide evidence regarding the diagnostic capacity of neuronal autoantibodies in irAE-n, but scarcely regarding their value to predict irAE-n. As the identification of patients at risk is particularly important, long-term and multicenter studies are warranted to further investigate the significance of preexisting subclinical autoimmune conditions in irAE-n.

## Conclusion

Neuromuscular autoantibodies may serve as a feasible marker to diagnose and potentially predict ICI-induced myositis, myocarditis, and myasthenia gravis. In contrast, brain-reactive autoantibodies are common in both ICI-treated patients with and without irAE-n, hence, their pathogenic significance needs further investigation.

## Data availability statement

The original contributions presented in the study are included in the article/**Supplementary Material**. Further inquiries can be directed to the corresponding author.

## Ethics statement

The studies involving human participants were reviewed and approved by Institutional ethics Committee Charité Universitätsmedizin Berlin. The patients/participants provided their written informed consent to participate in this study (EA1/ 099/17 and EA4/219/21).

## Author contributions

LMJ, SK, WB, PH and ME contributed to the conception and design of the study. LMJ, LGR, CS, SM, SK, CU, and RM enrolled the participants. LMJ, CS, SM, and LGR processed the blood samples, KR performed the experimental analysis. LMJ and PH performed the statistical analysis. LMJ wrote the first draft of the manuscript. LMJ, SK, WB, PH and ME acquired funding. All authors contributed to manuscript revision, read, and approved the submitted version.

## Glossary

**Table:**

ab	autoantibody
AchR	acetylcholine receptor
AMPAR 1/2	α-amino-3-hydroxy-5-methyl-4-isoxazolepropionic acid receptor 1/2
ANNA	anti-neuronal nuclear antibodies
AQP4	aquaporin 4
ARHGAP26	RhoGTPase-activating protein 26
CARP VIII	carbonic anhydrase related proteins VIII
CASPR2	contactin-associated protein-like 2
CBA	cell-based assays
CNS	central nervous system
CRMP5	collapsin response-mediator protein 5
CSF	cerebrospinal fluid
CTCAE	Common Terminology Criteria for Adverse Events
CTLA-4	cytotoxic T-lymphocyte-associated protein 4
DPPX	dipeptidyl-peptidase-like protein 6
GABA_A_R	gamma-aminobutyric-acid A receptor
GABA_B_R	gamma-aminobutyric-acid B receptor
GAD65	glutamic acid decarboxylase 65
GBS	Guillain-Barré
	
GFAP	glial fibrillary acidic protein
GluD2	glutamate receptor delta 2
GlyR	glycine receptor
Homer-3	Homer protein homolog 3
ICH	immunohistochemistry
ICI	immune checkpoint inhibitor
Ig	immunoglobulin
IgLON5	immunoglobulin LON5
IQR	interquartile range
irAE-n	neurological immune-related adverse events
irAEs	immune-related adverse events
ITPR-1	inositol 1,4,5-trisphosphate receptor 1
LGI1	leucine-rich glioma-inactivated 1
LRP4	lipoprotein receptor-related protein 4
MAG	myelin-associated glycoprotein
MG	myasthenia gravis
mGluR1	metabotropic glutamate receptor 1
mGluR5	metabotropic glutamate receptor 5
MOG	myelin oligodendrocyte glycoprotein
MRI	magnetic resonance imaging
MuSK	muscle-specific tyrosine kinase
NMDAR	N-methyl-D-aspartate receptor
No	number
NSCLC	non-small cell lung cancer
P/Q VGCC	P/Q-type voltage-gated calcium channel
PCA-1	purkinje cell cytoplasmic antibody 1
PD-1	programmed cell death protein 1
PD-L1	programmed death-ligand 1
PND	paraneoplastic neurological disorders
PNS	peripheral nervous system
RyR	ryanodine receptor
SOX1	SRY-related HMG-box 1
vs.	versus
Zic4	zinc finger 4
