# Supplementary material for: Autoantibody profiles in patients with immune checkpoint inhibitor-induced neurological immune related adverse events
Source: Front Immunol. 2023 Feb 8;14:1108116. doi: 10.3389/fimmu.2023.1108116 (PMC9945255; doi:10.3389/fimmu.2023.1108116)
Supplement: Supplementary file 1 [file DataSheet_1.docx]

Supplementary Material

*Original research*

**Autoantibody profiles in patients with immune checkpoint inhibitor-induced neurological immune related adverse events**

**Leonie Müller-Jensen^1,2^, Samuel Knauss^1,2^, Lorena Ginesta Roque^1^, Christian Schinke^1,2^, Smilla K Maierhof^1,2^, Frederik Bartels^1,2^, Carsten Finke^1,2,3^, Kristin Rentzsch^4^, Claas Ulrich^5^, Raphael Mohr^6^, Werner Stenzel^7^, Matthias Endres^1,8,9,10,11†^, Wolfgang Boehmerle^1,2,8†*^ and Petra Huehnchen^1,2,8†^**

^*^ Corresponding Author:

Wolfgang Boehmerle

Klinik für Neurologie mit Experimenteller Neurologie

Charité Universitätsmedizin Berlin

Charitéplatz 1, 10117 Berlin

Tel. +49 30 450 560137

Fax. +49 30 450 560932

Email: [wolfgang.boehmerle@charite.de](mailto:wolfgang.boehmerle@charite.de)

**Supplemental Table 1. Overview of all tested brain-reactive and neuromuscular autoantibodies.** Modified after Graus et al.^1^, Shelly et al.^2^ and Cetin et al.^3^

| **Antigen (alternative name)** | **Type** | **Association**  **with cancer** | **Associated disease** | **Epitope location** |
| --- | --- | --- | --- | --- |
| AMPAR1/2 | brain-reactive | intermediate | limbic encephalitis | surface |
| AQP4 | brain-reactive | low | neuromyelitis optica spectrum disorder | surface |
| ATP1A3 | brain-reactive | unknown, possibly high | cerebellar ataxia, brainstem encephalitis^4^ | surface |
| CASPR2 | brain-reactive | low /  intermediate | limbic encephalitis, Isaac syndrome,  Morvan’s syndrome | surface |
| DPPX | brain-reactive | low | encephalitis with CNS hyperexcitability  and PERM | surface |
| Flotillin1/2 | brain-reactive | unknown, possibly low | multiple sclerosis^5^ | surface |
| GABA_A_R | brain-reactive | low | encephalitis | surface |
| GABA_B_R | brain-reactive | intermediate | limbic encephalitis | surface |
| GluD2 | brain-reactive | high | cerebellar ataxia, encephalitis  opsoclonus-myoclonus syndrome | surface |
| GlyR | brain-reactive | low | limbic encephalitis, progressive  encephalomyelitis with rigidity  and myoclonus (PERM) | surface |
| IgLON5 | brain-reactive | unknown, possibly low | IgLON5-associated disease^6^ | surface |
| LGI1 | brain-reactive | low | limbic encephalitis | surface |
| mGluR1 | brain-reactive | low | rapidly progressive cerebellar syndrome^7^ | surface |
| mGluR5 | brain-reactive | intermediate | encephalitis | surface |
| MOG | brain-reactive | low | MOG antibody-associated disease | surface |
| Myelin | brain-reactive | unknown | unknown significance | surface |
| Neuro-endothelium | brain-reactive | unknown | unknown significance | surface |
| Neurexin | brain-reactive | unknown | encephalitis^8^ | surface |
| NMDAR IgG | brain-reactive | intermediate | anti-NMDAR encephalitis | surface |
| NMDAR IgA/IgM | brain-reactive | unknown | unknown significance^9^ | surface |
| Septin complex | brain-reactive | unknown | cerebellar ataxia | surface |
| Amphiphysin | brain-reactive | high | polyradiculoneuropathy, encephalomyelitis sensory neuronopathy, stiff-person syndrome | intracellular |
| ARHGAP26 | brain-reactive | unknown, possibly intermediate | cerebellar ataxia, psychosis,  cognitive impairment^10^ | intracellular |
| CARP VIII | brain-reactive | unknown, but association reported | rapidly progressive cerebellar syndrome^11^ | intracellular |
| CV2/CRMP5 | brain-reactive | high | sensory neuronopathy, encephalomyelitis | intracellular |
| **Antigen (alternative name)** | **Type** | **Association**  **with cancer** | **Associated disease** | **Epitope location** |
| GAD65 | brain-reactive | low | limbic encephalitis, stiff-person syndrome, cerebellar ataxia | intracellular |
| GFAP | brain-reactive | low | GFAP astrocytopathy^12^ | intracellular |
| Homer-3 | brain-reactive | unknown | cerebellar ataxia | intracellular |
| Hu (Anna-1) | brain-reactive | high | sensory neuronopathy, encephalomyelitis,  limbic encephalitis | intracellular |
| ITPR-1 | brain-reactive | unknown | cerebellar ataxia, epilepsy,  myelopathy, neuropathy | intracellular |
| Ma2 | brain-reactive | high | limbic encephalitis, diencephalitis,  brainstem encephalitis | intracellular |
| Neuro-chondrin | brain-reactive | unknown, possibly low | rhombencephalitis | intracellular |
| Recoverin | brain-reactive | unknown, possibly high | retinopathy^13^ | intracellular |
| Ri (Anna-2) | brain-reactive | high | brainstem/cerebellar syndrome | intracellular |
| Tr (DNER) | brain-reactive | high | rapidly progressive cerebellar syndrome | intracellular |
| Yo (PCA-1) | brain-reactive | high | rapidly progressive cerebellar syndrome | intracellular |
| Zic4 | brain-reactive | high | rapidly progressive cerebellar syndrome^14^ | intracellular |
| AchR | neuromuscular | associated with thymoma | myasthenia gravis | surface |
| LRP4 | neuromuscular | no | myasthenia gravis^15^ | surface |
| MAG | neuromuscular | high (MGUS) | Demyelinating neuropathy^16^ | surface |
| MuSK | neuromuscular | no | myasthenia gravis^17^ | surface |
| P/Q VGCC | neuromuscular | intermediate | Lambert-Eaton-myasthenic syndrome,  rapidly progressive cerebellar syndrome | surface |
| Heart muscle | neuromuscular | no | myocarditis, cardiomyopathies^18^ | intracellular |
| RyR | neuromuscular | associated with thymoma | myasthenia gravis | intracellular |
| Skeletal muscle | neuromuscular | associated with thymoma | myasthenia gravis^19^ | intracellular |
| SOX1 | neuromuscular | high | Lambert-Eaton-myasthenic syndrome with or without rapidly progressive cerebellar syndrome | intracellular |
| Titin | neuromuscular | associated with thymoma | myasthenia gravis | intracellular |

High = > 70% associated with cancer; intermediate = 30-70% associated with cancer; low = < 30% associated with cancer. AchR = acetylcholine receptor; AMPAR = α-amino-3-hydroxy-5-methyl-4-isoxazolepropionic acid receptor; ANNA = antineuronal nuclear antibody; AQP4 = aquaporin 4; ARHGAP26 = RhoGTPase-activating protein 26; CARP = carbonic anhydrase related proteins; CASPR2 = contactin-associated protein-like 2; CRMP5 = collapsin response-mediator protein 5; DNER = delta/ notch-like epidermal growth factor-related receptor; DPPX = dipeptidyl-peptidase-like protein 6; GABA_A_R = gamma-aminobutyric-acid A receptor; GABA_B_R = gamma-aminobutyric-acid B receptor; GAD65 = glutamic acid decarboxylase 65; GFAP = glial fibrillary acidic protein; GluD2 = glutamate receptor delta 2; GlyR = glycin receptor; Homer-3 = Homer protein homolog 3; Ig = immunoglobulin; ITPR1 = inositol 1,4,5-trisphosphate receptor 1; LGI1 = leucine-rich glioma-inactivated 1; LRP4 = lipoprotein receptor-related protein 4; MAG = myelin-associated glycoprotein; mGluR = metabotropic glutamate receptor; MGUS = monoclonal gammopathy of undetermined significance; MOG = Myelin oligodendrocyte glycoprotein; MuSK = muscle-specific tyrosine kinase; NMDAR = N-methyl-D-aspartate receptor; PCA = Purkinje cell antibody; P/Q VGCC = P/Q-type voltage-gated calcium channel; RyR = ryanodine receptor; SOX1 = SRY-related HMG-box 1; Zic4 = zinc finger 4.

1. Graus F, Vogrig A, Muñiz-Castrillo S, et al. Updated Diagnostic Criteria for Paraneoplastic Neurologic Syndromes. Neurol Neuroimmunol Neuroinflamm. 2021 May 18;8(4):e1014. doi: 10.1212/NXI.0000000000001014.

2. Shelly S, Kryzer TJ, Komorowski L, et al. Neurochondrin neurological autoimmunity. *Neurol Neuroimmunol Neuroinflamm.* 2019 Sep 11;6(6):e612. doi: 10.1212/NXI.0000000000000612.

3. Cetin H, Vincent A. Pathogenic Mechanisms and Clinical Correlations in Autoimmune Myasthenic Syndromes. *Semin Neurol.* 2018 Jun;38(3):344-354. doi: 10.1055/s-0038-1660500.

4. Scharf M, Miske R, Heidenreich F, et al. Neuronal Na+/K+ ATPase is an autoantibody target in paraneoplastic neurologic syndrome. *Neurology.* 2015 Apr 21;84(16):1673-9. doi:10.1212/WNL.0000000000001493.

5. Hahn S, Trendelenburg G, Scharf M, et al. Identification of the flotillin-1 / 2 heterocomplex as a target of autoantibodies in bona fide multiple sclerosis. *J Neuroinflammation.* 2017 Jun 23;14(1):123. doi: 10.1186/s12974-017-0900-z.

7. Sabater L, Gaig C, Gelpi E, et al. A novel non-rapid-eye movement and rapid-eye-movement parasomnia with sleep breathing disorder associated with antibodies to IgLON5 : a case series, characterisation of the antigen, and post-mortem study. *Lancet Neurol.* 2014 Jun;13(6):575-86. doi: 10.1016/S1474-4422(14)70051-1.

8. Sillevis Smitt P, Kinoshita A, De Leeuw B, et al. Paraneoplastic cerebellar ataxia due to autoantibodies against a glutamate receptor. *N Engl J Med.* 2000 Jan 6;342(1):21-7. doi: 10.1056/NEJM200001063420104.

9. Gresa-Arribas N, Planagumà J, Petit-Pedrol M, et al. Human neurexin-3 a antibodies associate with encephalitis and alter synapse development. *Neurology*. 2016;86(24):2235-2242.

10. Hara M, Martinez-Hernandez E, Ariño H, et al. Clinical and pathogenic significance of IgG, IgA, and IgM antibodies against the NMDA receptor. Neurology. 2018 Apr 17;90(16):e1386-e1394. doi: 10.1212/WNL.0000000000005329

10. Bartels F, Prüss H, Finke C. Anti-ARHGAP26 Autoantibodies Are Associated With Isolated Cognitive Impairment. *Front Neurol.* 2018 Aug 10;9:656. doi: 10.3389/fneur.2018.00656.

11. Höftberger R, Sabater L, Velasco F, et al. Carbonic Anhydrase-Related Protein VIII antibodies and paraneoplastic cerebellar degeneration. *Neuropathol Appl Neurobiol*. 2014;40(5):650-653. doi:10.1111/nan.12118.

13. Gravier-Dumonceau A, Ameli R, Rogemond V, et al. Glial Fibrillary Acidic Protein Autoimmunity. *Neurology*. 2022;98(6):e653-e668. doi:10.1212/WNL.0000000000013087

14. Bataller L, Wade DF, Graus F, et al. Antibodies to Zic4 in paraneoplastic neurologic disorders and small-cell lung cancer. *Neurology.* 2004 Mar 9;62(5):778-82. doi: 10.1212/01.wnl.0000113749.77217.01

15. Zisimopoulou P, Evangelakou P, Tzartos J, et al. A comprehensive analysis of the epidemiology and clinical characteristics of anti-LRP4 in myasthenia gravis. *J Autoimmun*. 2014 Aug;52:139-45. doi: 10.1016/j.jaut.2013.12.004.

16. Ellie E, Steck A, Intgrative N. Neuropathy associated with "benign" anti-myelin-associated glycoprotein IgM gammopathy: clinical, immunological, neurophysiological pathological findings and response to treatment in 33 cases. *J Neurol.* 1996 Jan;243(1):34-43. doi: 10.1007/BF00878529.

17. Lauriola L, Ranelletti F, Maggiano N, et al. Thymus changes in anti-MuSK-positive and -negative myasthenia gravis. *Neurology.* 2005 Feb 8;64(3):536-8. doi: 10.1212/01.WNL.0000150587.71497.B6.

18. Caforio AL, Mahon NJ, Tona F, McKenna WJ. Circulating cardiac autoantibodies in dilated cardiomyopathy and myocarditis: pathogenetic and clinical significance. *Eur J Heart Fail.* 2002 Aug;4(4):411-7. doi: 10.1016/s1388-9842(02)00010-7.

19. Romi F, Skeie GO, Gilhus NE AJ. Striational Antibodies in Myasthenia Gravis Reactivity and possible clinical significance. *Arch Neurol*. 2005 Mar;62(3):442-6. doi: 10.1001/archneur.62.3.442.

**Supplemental Table 2.** **Clinical characteristics and neuronal autoantibody profiles of patients with ICI-induced neurological immune related adverse events**

| **No.** | **Sex** | **Age** | **Tumor** | **ICI** | **IrAE-n** | **Time from ICI start to onset (weeks)** | **Worst CTCAE grade of irAE-n** | **CSF** | **Neuronal autoantibodies** | **Additional irAE** | **Treatment of**  **irAE-n** | **Neurological outcome**  **(CTCAE at 3m after onset)** | **Steroids**  **at 3m after**  **onset** | **ICI rechallenge** | **Tumor outcome at 3m after onset** |
| --- | --- | --- | --- | --- | --- | --- | --- | --- | --- | --- | --- | --- | --- | --- | --- |
| 1 | m | 75 | UC | PEM | hypophysitis (CNS), neuropathy (PNS) | 16 | 3 | 3 cells / μl, protein ↑ | none^a^ | none | i.v. steroids (250mg/d), then oral taper | 2 (at one month after onset) | NA | no | NA |
| 2 | f | 63 | MM | NIV  (before  IPI  + NIV) | encephalitis (CNS),  myositis (PNS) | 37 | 3 | 6 cells / μl, protein ↑ | anti-skeletal muscle 1:40 | dematitis, hepatitis, exanthema, thyreoiditis,  myocarditis pneumonitis | oral steroids (70mg/d prednisolone),  then taper | 2 | yes | NA | SD |
| 3 | m | 77 | MM | PEM  (before  IPI  + NIV) | myositis (PNS), neuropathy (PNS), earlier: encephalitis (CNS) | 6 | 4 | 211 cells / μl, protein ↑↑ | anti-heart muscle positive, anti-skeletal muscle 1:80, anti-AchR 0.5 nmol/l,  anti-titin ++ | myocarditis, thyreoditis,  pancreatitis | i.v. steroids (1g/d),  then oral taper; IVIG | 2 | yes | yes, relapse of myositis | PR |
| 4 | m | 81 | MM | IPI+ NIV  (before: NIV mono) | encephalitis (CNS) | 18 | 4 | 51 cells / μl, protein ↑↑, neuronal abs negative | anti-GFAP 1:320 | pneumonitis | i.v. steroids (1g/d),  then oral taper | 2 | yes | NA | NA |
| 5 | f | 77 | MM | IPI + NIV | encephalitis (CNS) | 3 | 5 | 12 cells / μl, protein ↑, neuronal abs negative | none | hepatitis | i.v. steroids (1g/d), plasma exchange | 5 | NA | no | NA (died) |
| 6 | m | 68 | MM | NIV  (before  IPI  + NIV) | hypophysitis (CNS), neuropathy (PNS) | 24 | 2 | NA | anti-GABA_B_R 1:32 | thyreoiditis, nephritis, dermatitis | oral steroids (70mg/d prednisolone),  then taper | 2 | yes | no | SD |
| 7 | m | 56 | CC | PEM | encephalitis (CNS) | 25 | 3 | 21 cells / μl, protein ↑↑; neuronal abs negative | anti-NMDAR IgA 1:10 | none | i.v. steroids (1g/d),  then taper | 2 (at one month after onset) | yes | NA | PD |
| **No.** | **Sex** | **Age** | **Tumor** | **ICI** | **IrAE-n** | **Time from ICI start to onset (weeks)** | **Worst CTCAE grade of irAE-n** | **CSF** | **Neuronal autoantibodies** | **Additional irAE** | **Treatment of**  **irAE-n** | **Neurological outcome**  **(CTCAE at 3m after onset)** | **Steroids**  **at 3m after onset** | **ICI rechallenge** | **Tumor outcome at 3m after onset** |
| 8 | m | 58 | NSCLC | PEM | encephalitis (CNS) | 59 | 4 | 34 cells / μl, protein ↑↑,  neuronal abs negative | anti-RyR positive | myocarditis | i.v. steroids (1g/d),  then oral taper | 2 | yes | no | PR |
| 9 | f | 61 | GC | NIV | encephalitis (CNS) | 12 | 4 | 5 cells / μl, protein normal, lactate ↑, neuronal abs negative | anti-NMDAR IgA 1:320  anti-NMDAR IgM 1:10 | hepatitis | i.v. steroids (1g/d) | last CTCAE: 4 (died of sepsis one week after irAE-n onset) | NA | no | NA (died) |
| 10 | f | 68 | MM | IPI  +/- NIV^b^ | hypophysitis (CNS) | 24 | 3 | NA | none^a^ | thyreoiditis | hydrocortison | 2 | yes | no | SD |
| 11 | f | 86 | MM | NIV | myositis (PNS) | 3 | 5 | 3 cells / μl, protein ↑,  neuronal abs negative | anti-heart muscle positive,  anti-skeletal muscle 1:160,  anti-titin +++  (abs positive before and after ICI treatment!) | myocarditis | i.v. steroids (1g/d) | 5 | NA | no | NA (died) |
| 12 | m | 79 | MM | IPI + NIV | hypophysitis (CNS), myositis (PNS) | 3 | 4 | 12 cells / μl, protein ↑↑,  neuronal abs negative  (pleocytosis due to spondylo-discitis) | anti-AchR 3.4 nmol/l, anti-LRP4 positive | myocarditis, exanthema | i.v. steroids (1g/d),  then oral taper; IVIG | 3 | yes | no | SD |
| 13 | m | 58 | NSCLC | NIV | myositis / myopathy (PNS) | 44 | 3 | NA | anti-RyR borderline positive | none | oral steroids (70mg/d prednisolone),  then taper | 3 | yes | NA | NA |
| 14 | m | 69 | MM | IPI + NIV | neuropathy (PNS) | 12 | 3 | NA | none^a^ | pruritus | oral steroids (80mg/d prednisolone),  then taper | 2 | yes | ICI continued | PD |
| **No.** | **Sex** | **Age** | **Tumor** | **ICI** | **IrAE-n** | **Time from ICI start to onset (weeks)** | **Worst CTCAE grade of irAE-n** | **CSF** | **Neuronal autoantibodies** | **Additional irAE** | **Treatment of irAE-n** | **Neurological outcome**  **(CTCAE at 3m after onset)** | **Steroids at 3m after onset** | **ICI rechallenge** | **Tumor outcome at 3m after onset** |
| 15 | m | 62 | MM | NIV | myositis, GBS-like polyradiculitis (PNS) | 15 | 3 | NA | anti-heart muscle positive | thyreoiditis,  hepatitis | i.v. steroids (1g/d),  then oral taper | 3 | yes | yes | PD |
| 16 | m | 33 | HL | NIV | neuropathy / GBS-like polyradiculitis (PNS) | 20 | 3 | 10 cells / μl, protein ↑↑ | none^a^ | none | IVIG (2g/kg) | 3 | no | no | PD |
| 17 | f | 61 | MM | NIV | neuropathy (PNS) | 6 | 2 | NA | anti-myelin (CNS)^a^  1:100 | none | oral steroids | 2 | yes | ICI continued | PD |
| 18 | m | 78 | MM | NIV | exacerbation of preexisting myasthenia gravis (PNS) | 1 | 4 | NA | anti-titin +,  anti-AchR positive^c^ | none | i.v. steroids (100mg/d prednisolone), IVIG, plasma exchange | 3 | yes | no | PD |
| 19 | m | 71 | PC | PEM | ocular myasthenia (PNS) | 7 | 2 | NA | anti-titin +,  anti-skeletal muscle 1:160, anti-heart muscle positive | myocarditis | oral steroids (50mg/d prednisolone), then taper; pyridostigmin | 2 | yes | no | PR |
| 20 | m | 61 | HCC | ATE | myositis (PNS) | 9 | 5 | 2 cells / μl, protein normal,  neuronal abs negative | anti-NMDAR IgG 1:32,  anti-titin +++,  anti-skeletal muscle 1:160, anti-heart muscle positive, anti-RyR positive | none | i.v. steroids (1g/d) | 5 | NA | no | NA (died) |
| 21 | f | 67 | MM | NIV | myositis (PNS) | 57 | 3 | NA | anti-septin complex 1:320, IH with anti-neuroendothelial abs | colitis,  hepatitis | oral steroids (10mg/d prednisolone), then taper | 2 | yes | no | CR |
| 22 | m | 74 | MM | PEM | myositis,  myasthenia gravis (PNS) | 1 | 3 | NA | IH with brain-reactive abs of unknown reactivity; anti-heart muscle positive,  anti-skeletal muscle 1:160 | myocarditis | oral steroids (2mg/kg prednisolone), then taper; IVIG (2g/kg) | 3 | yes | yes, relapse of myositis / myasthenia | SD |
| **No.** | **Sex** | **Age** | **Tumor** | **ICI** | **IrAE-n** | **Time from ICI start to onset (weeks)** | **Worst CTCAE grade of irAE-n** | **CSF** | **Neuronal autoantibodies** | **Additional irAE** | **Treatment of irAE-n** | **Neurological outcome**  **(CTCAE at 3m after onset)** | **Steroids at 3m after onset** | **ICI rechallenge** | **Tumor outcome at 3m after onset** |
| 23 | m | 58 | MM | PEM | hypophysitis (CNS),  neuropathy (PNS) | 23 | 2 | NA | anti-NMDAR IgM 1:100,  anti-AchR 0.4 nmol/l (borderline) | none | oral steroids (80mg/d prednisolone), then taper | 2 | yes | NA | NA |
| 24 | m | 65 | SCLC | ATE | myopathy, dropped head syndrome (PNS) | 11 | 3 | 5 cells / μl, protein normal,  neuronal abs negative | none | none | i.v. steroids (1g/d), then oral taper | 3 | yes | no | PR |
| 25 | m | 56 | OGJC | IPI + NIV | neuropathy (PNS) | 7 | 3 | NA | none | none | i.v. steroids (1g/d), then oral taper; IVIG | 3 | yes | no | PR |
| 26 | m | 72 | HCC | ATE | neuropathy, myasthenia gravis (PNS) | 52 | 2-3 | NA | anti-NMDAR IgM 1:3200,  anti-RyR borderline positive | arthritis | oral steroids (1mg/kg prednisolone), then taper; IVIG | 2 | yes | no | PR |
| 27 | m | 61 | MCC | AVE | myopathy, neuropathy (PNS) | 156 | 2 | NA | anti-NMDAR IgM 1:100,  anti-NMDAR IgA 1:32 | none | no treatment | 2 | no | ICI continued | SD |
| 28 | m | 61 | SCLC | ATE | GBS (PNS) | 10 | 3 | 22 cells / μl, protein ↑↑↑ | anti-GABA_B_R 1:10,  anti-Zic4 1:32000,  anti-RyR positive,  anti-LRP4 positive | polyarthritis | i.v. steroids (500mg/d), then taper, IVIG (2g/kg) | 3 | yes | no | NA |
| 29 | f | 52 | MM | IPI + NIV | peripheral facial nerve palsy (PNS) | 8 | 2 | NA | before ICI treatment:  anti-NMDAR IgG 1:100  after ICI treatment:  anti-AchR 0.6 nmol/l  anti-GABA_B_ 1:10  anti-NMDAR IgG 1:10 | hepatitis,  exanthema | oral steroids (60mg/d prednisolone), then taper | 1 | yes | NA | PR |

Ab = autoantibody; AchR = acetylcholine receptor; ATE = atezolizumab; AVE = avelumab; CCA = cholangiocarcinoma; CR = complete remission; CTCAE = Common Terminology Criteria for Adverse Events; f = female; GABA_B_R = gamma-aminobutyric-acid A receptor; GBS = Guillain-Barré syndrome; GC = gastric cancer; GFAP = glial fibrillary acidic protein; HCC = hepatocellular carcinoma; HL = Hodgkin lymphoma; ICI = immune checkpoint inhibitor; IH = immunohistochemistry; IPI = ipilimumab; irAE = immune related adverse event; IVIG = intravenous immunoglobulin therapy; LRP4 = lipoprotein receptor-related protein 4; m = male; MCC = Merkel cell carcinoma; MM = malignant melanoma; NA = no data available; NIV = nivolumab; NMDAR = N-methyl-D-aspartate receptor; No. = number; NSCLC = non-small cell lung cancer; OGJA = oesophagogastric junctional adenocarcinoma; PD = progressive disease; PEM = pembrolizumab; PR = partial remission; RyR = ryanodine receptor; SCLC = small cell lung cancer; SD = stable disease; UC = urothelial carcinoma. Zic4 = Zinc finger protein 4; 3m = three months; + = slightly positive; ++ = moderately positive; +++ = highly positive. ^a^ = in these patients only brain-reactive antibodies were tested. ^b^ = treatment was blinded for either nivolumab-monotherapy or combination therapy of nivolumab + ipilimumab ^c^ = in this patient the following autoantibodies could not be measured: anti-MuSK, anti-RyR, anti-LRP4, anti-skeletal muscle, anti-heart muscle.

**Supplemental Table 3.** **Comparison of demographics between irAE-n patients with and without neuronal autoantibodies**

|  | **p-values** | | **p-values** | |
| --- | --- | --- | --- | --- |
| **Variable** | **Comparison of irAE-n patients with and without neuromuscular autoantibodies** | **Test** | **Comparison of irAE-n patients with and without brain-reactive autoantibodies** | **Test** |
| Sex | 0.64 | Fisher's exact test | 1 | Fisher's exact test |
| Age | 0.72 | Unpaired Student's t-test | 0.2 | Unpaired Student's t-test |

IrAE-n = neurological immune related adverse events.
